# Comparative effects of two *in situ* hybridization methods for the pinewood nematode (*Bursaphelenchus xylophilus*)

**DOI:** 10.3389/fmicb.2023.1234895

**Published:** 2023-11-30

**Authors:** Chunyu Wang, Junhao Zhuge, Siqi Tang, Xiang Zhou, Lifeng Zhou, Kai Guo

**Affiliations:** ^1^School of Forestry and Biotechnology, Zhejiang A&F University, Hangzhou, China; ^2^National Joint Local Engineering Laboratory for High-Efficient Preparation of Biopesticide, Changsha, China

**Keywords:** *in situ* hybridization, cut-off ISH, whole-mount ISH, pinewood nematode, method

## Abstract

The gene localization technique of *Bursaphelenchus xylophilus* (pinewood nematode, PWN) is used for study gene expression in PWNs. Two *in situ* hybridization methods, namely, whole-mount *in situ* hybridization and the cut-off method are used widely. To compare the effects of these two *in situ* hybridization methods, the present study investigated the patterns of two functional genes expression in PWNs. The *Bx-vap-2* gene (GenBank accession number: OR228482), related to pathogenicity, and the *fem-2* gene (GenBank accession number: OR228481), related to sex determination, were selected to map related genes in the whole-mount and amputated PWNs at different ages using these *in situ* hybridization methods. Based on the overall statistical comparison, we found that compared to the cut-off method, the whole-mount method exhibited higher staining rates and correct staining rates for the *fem-2* gene and *Bx-vap-2* gene. However, considering the correct staining aspect, the cut-off method yielded better staining effects on pinewood nematode sections than the whole-mount method, with clearer hybridization signal locations and less non-specific staining. In other words, the cut-off method demonstrated more precise gene localization. Both methods are applicable for gene localization, but considering the overall staining pattern, analysis of experimental results, and comprehensive experimental operations, we believe that the whole-mount method is more suitable for gene localization and expression analysis of development-related genes in pinewood nematodes. This is because intact pinewood nematodes are better suited for showcasing the continuous developmental process of development-related genes. On the other hand, considering the experimental time, accuracy of staining site, and the amount of non-specific staining, the cut-off method is more suitable for disease-related genes. Additionally, to achieve better performance, the cut-off method can be selectively applied to samples during the experimental process.

## 1 Introduction

Pine wilt disease (PWD) is caused by pinewood nematode (PWN, *Bursaphelenchus xylophilus*) and is a highly dangerous and devastating epidemic disease in forests (Mota and Vieira, 2008; Jeong et al., 2013). PWNs invade, feed, and multiply rapidly in pine trees. And pine trees will wilt and die rapidly in a short period; hence, it is also referred to as pine cancer (Allison et al., 2012). PWD has widely spread in 20 provinces in China, with an annual death of millions of pine trees, causing serious economic and ecological losses; therefore, it is classified as the most important forestry pest quarantine target in China (Mota et al., 1990; Khan and Gbadegesin, 1991).

Researches on PWN have focused on understanding its pathogenicity and biological properties, and on elaborating the function and expression of genes at the molecular level, the *in situ* hybridization method is widely applied for determining the spatiotemporal expression of target genes throughout the life cycle of PWNs (Zhou et al., 2021). The *in situ* hybridization is based on the principle of complementary base pairing of nucleic acid molecules, deploying a radiolabeled or non-radiolabeled nucleic acid probe that is complementary to denatured RNA or DNA to form a specific nucleic acid hybrid molecule, and displaying the position of the nucleic acid on the cell, tissue, or chromosome using certain staining methods (Lin et al., 2017). As the nematode body is transparent, the probed nucleic acid hybrid molecules can be clearly observed under a microscope, making it an ideal technique for studying the spatiotemporal expression of the nematodes’ genes.

There are two *in situ* hybridization methods, including the whole-mount *in situ* hybridization and the cut-off method, used widely in nematology. Since De Boer et al. developed a method of cutting second-stage larvae in diluted formaldehyde fixative and immediately using them for hybridization to reduce mRNA degradation and improve probe penetration into tissues (De Boer et al., 1998). This method was later applied in the pine wood nematode as well. Espada et al. (2016) used *in situ* hybridization technique to investigate the spatial expression patterns of the 46 putatively secreted proteins in mixed life stage nematodes. Kikuchi et al. (2004) modified the digoxigenin RNA probe used in the cut-off method to DIG (digoxigenin)-labeled sense or antisense DNA probes for the localization analysis of the *Bx-eng-1* gene in the pine wood nematode. Subsequently, this method was used for the localization of the *Bxy-ctl-1* and *Bxy-ctl-2* genes in the pine wood nematode, and the localization of seven GH18 chitinase genes in pine wood nematode tissues (Vicente et al., 2015; Ju et al., 2016). Tang et al. (2020) applying whole-mount *in situ* hybridization and RNA probes to the pine wood nematode. They discovered that the *Bxy-fuca* gene is primarily expressed in the body wall muscles and intestine. Subsequently, Zhou et al. (2021) also successfully utilized whole-mount *in situ* hybridization for intact nematode using DIG-labeled single-stranded RNA (ss RNA) probes. Wen et al. (2021) applied the DIG-labeled antisense cDNA probes and the whole-mount *in situ* hybridization to the intact pine wood nematode and demonstrated that *BxSCD1* was specifically expressed in the dorsal glands and intestine of PWN.

However, the superiority of these two *in situ* hybridization methods has not been reported for PWNs. Therefore, in this study, the whole-mount method and the cut-off method were used to perform *in situ* hybridization on PWNs of different ages. The results were compared to determine the more suitable method for PWNs. To increase the scientific validity of the study, the experiment selected the pathogenicity-related *Bx-vap-2* gene and the sex-determining-related *fem-2* gene for testing. In order to compare the gene localization of two different probes, cDNA and ssRNA, under two different treatments involving intact and severed nematode bodies, we interchanged the reagents, probes, and experimental procedures of the two methods. This allowed us to observe whether the application of whole-mount *in situ* hybridization with the probe, reagents, and methods, along with the treatment of severed nematodes, resulted in a more efficient and accurate gene localization within the nematode bodies. Additionally, we examined the gene expression patterns using the severed nematode treatment with the probes, reagents, and methods employed in the severed method. It is expected that this experiment will contribute to the gene expression localization of PWNs, promote molecular biology-related research on PWNs.

## 2 Materials and methods

### 2.1 Experimental materials

#### 2.1.1 Test nematodes and culture conditions

The PWN samples were provided by the Institute of Forest Protection, Chinese Academy of Forestry. PWNs were subcultured on a *Botrytis cinerea* (gray mold) lawn on potato dextrose agar. Many PWNs of different ages were obtained by incubation at 25°C for 3 days in the dark. Nematodes were separated from the culture medium using Bellman funnels.

#### 2.1.2 Holistic and cut-off acquisition of synchronous-age nematodes

When a large number of females were in an egg-bearing state, nematodes were separated and added to sterilized double-distilled water (ddH_2_O), and eggs were laid in an incubator set at 25°C for 1 h. The nematode suspension was gently removed and washed once or twice, and synchronized nematode embryos were obtained by adding ddH_2_O to the Petri dishes. Synchronized second-stage juvenile (J2) larvae were obtained by adding ddH_2_O to the synchronized embryos and placing them in an incubator at 25°C for 24 h. Synchronized third and fourth parasitic stage (J3 and J4) larvae and adults were obtained by culturing synchronized J2 larvae on gray mold plates and incubating them at 25°C for 36, 48, and 60 h, respectively (Zhou et al., 2021).

Half of the synchronous-age nematodes were washed three times with 1 × phosphate-buffered saline (PBS) and fixed in 10% (v/v) formaldehyde diluted with 1 × PBS for 2 days at room temperature (25°C). Fixed nematodes were randomly cut on glass slides with a razor blade until approximately 90% of the nematodes were cut and prepared.

#### 2.1.3 Strains and plasmids

*Botrytis cinerea* (gray mold) was a kind gift from the Nematology Laboratory at Zhejiang University. The pGEM-T® Easy Vector system was purchased from Promega (Madison, Wisconsin, USA). TOP-10 competent cells (CB104) were purchased from TIANGEN Biotech (Beijing) Co., Ltd., China.

#### 2.1.4 Major reagents and instrumentation

1. Main reagents: TRIzol™ Reagent of Invitrogen (Shanghai); PrimeScript™ RT reagent Kit with gDNA Eraser (Perfect Real Time), Premix Taq™ (Ex Taq™ Version 2.0), 2000 DNA Marker of TaKaRa (Beijing); pGEM-Teasy kit, T7/SP6 RNA Polymerase, and Rnase inhibitor of Promega; DIG-labeling reagents and antibodies of ROCHE (Basel, Switzerland); Diethyl pyrocarbonate (DEPC), 50× TAE buffer, ampicillin, Tris, 4 s red plus nucleic acid dye, Dithiothreitol (DTT), Tween-20, N-2-hydroxyethylpiperazine-N-2-ethane sulfonic acid (HEPES), Ethylene Glycol Tetraacetic Acid (EGTA), Glycine, Dimethyl sulfoxide (DMSO), and other common reagents from Sangon Biotech (Shanghai). Primer synthesis and sequencing were carried out by Tsingke Biotechnology Co., Ltd.
2. Main equipment: TU-100 constant-temperature metal bath of Shanghai yiheng technology Co., Ltd., SHP-250 biochemical incubator of Shanghai Jinghong Experimental Equipment Co., Ltd., PHM-70 automatic high-pressure steam sterilizer of Shanghai Youngster Intelligence Technology Co., Ltd., DW-86L388J Medical Cryopreservation Box of Qingdao Special Electric Appliance Co., Ltd., ME1002E/02 digital balance of METTLER TOLEDO instrument Shanghai co., Ltd., Eq02520-300-rd000 precellys evolution by Bertin Technologies (Montigny-le-Bretonneux, France), ICEN-24R high-speed refrigerated centrifuge of Hangzhou Aosheng Instrument Co., Ltd., TC-XP-D Gene Amplifier of Hangzhou Bori Technology Co., Ltd., DYY-6C electrophoresis apparatus of Beijing Liuyi Biotechnology Co., Ltd., LG2020 Gel Imaging System of Hangzhou LongGene Scientific Instruments Co., Ltd., NanoDrop 2000 spectrophotometer of Thermo Scientific (Waltham, Massachusetts, USA), LF-IIIA hridization oven of Ningbo Xinzhi Biotechnology Co., Ltd., DM4 B microscope of Leica (Germany), and SZ650 continuous zoom stereomicroscope of Chongqing Aote Optical Instrument Co., Ltd.

### 2.2 Experimental methods

#### 2.2.1 Extraction and detection of total RNA from PWN

Total RNA was extracted using the TRIzol method following the procedures described by Liu et al. (2022).

Mix the reagents, magnetic beads, and nematodes in a 2 mL centrifuge tube, grind, and centrifuge. Combine the supernatant with chloroform in a new centrifuge tube, mix, and centrifuge. Combine the supernatant with isopropanol in a new centrifuge tube, mix, and centrifuge. Remove the supernatant, wash RNA with 75% ethanol, and centrifuge. Air-dry the RNA precipitate. Dissolve RNA in ribonuclease-free water.

#### 2.2.2 PWN RNA reverse transcription

After the RNA quality and concentration requirements were met, PWN cDNA was prepared using the TaKaRa Reverse Transcription Kit according to the manufacturer’s instructions. The present study involved reverse transcription experiments conducted on RNA samples with A260/A280 ratios between 1.8 and 2.0 and A260/A230 ratios approximately around 2.0; During the reverse transcription experiment, the amount of RNA used was 1 microgram.

#### 2.2.3 Acquisition of coding region of PWN *Bx-vap-2* and *fem-2* genes

1. The coding regions of homologous genes of PWN were predicted by analyzing the *Bx-vap-2* and *fem-2* genes of the model organism, *C. elegans*, and mapping them to the available genomic and transcriptomic databases of PWN. Primers were designed using Oligo 7 as follows:
  Bx-vap-F: 5′-GAACAATGCTGAACGGAACG-3′
  Bx-vap-R: 5′-TTGGACAAGCGGCTACGG-3′
  fem-F: 5′-GAAAGACTCGTTGTTCGCAGTA-3′
  fem-R: 5′-CGATTTGAGAAGCCGGTAG-3′
2. PCR amplification
  The PCR reaction mixture contained 25 μL Premix Taq, 1 μL cDNA, 1 μL each of the forward and reverse primers (10 μM), and 22 μL ddH_2_O to obtain a total system volume of 50 μL.
  The PCR amplification steps were as follows: denaturation at 94°C for 5 min; 35 cycles of 94°C for 30 s, 60°C for 30 s, and 72°C for 1 min; and extension at 72°C for 10 min. The PCR amplification products were detected by agarose gel electrophoresis and sent for sequencing.
3. The target amplicons were recovered using a Gel Recovery Kit (TaKaRa) and stored at −20°C.

#### 2.2.4 Construction of PWN *Bx-vap-2* and *fem-2* plasmid vectors

1. Construction of recombinant plasmids
  Purified gene amplicons were ligated to Promega pGEM-T Easy vectors overnight at 4°C according to the manufacturer’s instructions.
2. The recombinant plasmids were transformed into *Escherichia coli* competent cells and screened for recombinants, according to the method described by Wang et al. (2022). Colony PCR followed by gel electrophoresis was performed, and the plasmids were sent for sequencing and plasmid prep.

#### 2.2.5 Whole-mount method for PWN *Bx-vap-2* and *fem-2* whole-mount and cut-off *in situ* hybridization

1. Identification of target fragment insertion direction
  Four combinations of primers—GSR + M13R, GSR + M13F, GSF + M13R, and GSF + M13R—were used to amplify target fragments using the recombinant plasmid as a template to identify the direction of the target fragment insertion.
2. ssRNA probe preparation
  After the target gene insertion direction was determined, the target fragments containing the T7 and SP6 promoters were amplified by PCR using the plasmids as templates.
  - The reaction system containing 4 μL 5 × buffer, 0.5 μL Rnase inhibitor, 2 μL DTT (100 mM), 2 μL 10 × DIG RNA Labeling Mix, 1 μL RNA polymerase, and DNA Template x (Max: 10.5 μL, ≤ 1 μg) was mixed well and incubated in a warm bath at 37°C for 1 to 1.5 h (no more than 1.5 h).
  - DNA was removed by adding 2 μL Dnase I to the reaction tube and incubating the mixture at 37°C for 15 min.
  - The reaction was terminated by adding 2 μL of 200 mM EDTA (pH 8.0).
  - The probe was precipitated by adding 2.5 μL of 4 M lithium chloride and 75 μL of EtOH (pre-chilled at 4°C) and stored at −20°C overnight.
  - The pellet was washed by placing the tube in a high-speed refrigerated centrifuge at 13800 xg for 15 min at 4°C, removing the supernatant, adding 50 μL of 70% EtOH, flicking the tube, centrifuging again at 13800 xg for 8 min at 4°C, removing the supernatant, and letting the pellet stand at room temperature (25°C) with the cap open for 5 min to remove the remaining EtOH.
  - The probe was preserved by adding 50 μL Rnase-free water to dissolve the pellet, and then adding 1 μL Rnase inhibitor, making aliquots of 1 μg per tube, and storing at −80°C.

##### 2.2.5.1 Fixation of PWNs

1. Initial fixation of PWNs
  - Intact synchronized PWNs of all ages were washed in advance two to three times with DEPC water; sections did not need to be washed again.
  - One milliliter of 1 × BO_3_ (solvent: DEPC-treated H_2_O, containing 10 mM DTT and 0.1% v/v Tween-20) was added and the PWNs were placed in a hybridization oven rotating for 20 min at 22°C.
  - One milliliter of 1× PBS (pre-chilled at 4°C) was added and the PWNs were placed in a molecular hybridization oven for 2 min at 22°C; this was repeated once.
  - Enzyme K digestion: PBT (1 mL) containing 5 μL proteinase K (20 mg/mL) was added, and the PWNs were placed in a hybridization oven rotating for 30 min at 22°C.
  - Termination of digestion: 1 mL of pre-chilled glycine in PBT at 4°C was added and the sample was rotated for 2 min in a hybridization oven.
  - One milliliter of 1× PBS was added and the sample was placed in a hybridization oven rotating for 2 min at 22°C; this was repeated twice.
  - One milliliter of 4°C pre-chilled Dent (MeOH: DMSO = 8:2) was added, and the sample was left in an ice box for 5 min.
  - Transfer and replenishment process (based on 1 mL) (Supplementary Table S1) (The initially fixed nematodes can be temporarily stored in EtOH at −20°C.)
2. Fixation of PWNs
  - One milliliter of PBT was added and the sample was placed in a hybridization oven rotating for 5 min at room temperature (25°C); this was repeated twice.
  - Enzyme K digestion: PBT (1 mL) containing 0.333 μL proteinase K (20 mg/mL) was added and the sample was placed in a hybridization oven and rotated at 37°C for 30 min.
  - Termination of digestion: 1 mL of 4°C pre-chilled glycine in PBT was added and the sample was rotated for 2 min in a hybridization oven.
  - Acetylation: 1 mL of 0.1% triethanolamine was added, and the sample was rotated for 2 min at room temperature (25°C). Then, 1 mL of 0.05% acetic anhydride in triethanolamine was added, and the sample was rotated for 10 min at room temperature (25°C).

##### 2.2.5.2 Hybridization and detection

1. Fluid change per 1 mL (Supplementary Table S2)
2. Pre-hybridization: 2.0 mL → 250 μL/well
  Hybridization buffer (2 mL) was placed in a 2-mL Rnase-free centrifuge tube, which was then placed in a water bath at 99°C for 10 min for denaturation, followed by a rapid ice bath for 5 min, mixed well with PWN, and aliquoted evenly into 8-well plates ensuring approximately 300 intact nematodes or 500 nematode sections per well, with three replicates at each age. Eight-well plates were placed in a humidity gasket and incubated in a hybridization oven at 48°C for 1 h.
3. Hybridization: 2.0 mL → 250 μL/well
  Briefly, 250 μL of hybridization buffer was added to 1.5-mL Rnase-free centrifuge tubes, and 1 μg of the probe was added to each tube, which was then placed in a water bath at 99°C for 10 min of denaturation, followed by a rapid ice bath for 5 min, and then transferred to a corresponding 8-well plate, which was then placed in a humidity gasket in a hybridization oven at 48°C for approximately 16–18 h.
4. Washing: 1.8 mL → 200 μL/well
  - Solution 1 (50% formamide, 5× SSC, 100 μg/mL heparin, 0.1% Tween,PBT = 1,1) and solution 2 (0.8× PBS, 0.1% CHAPS) were prepared and pre-heated to 48°C.
  - Pre-heated solution 1 (200 μL, 48°C) was added to each well, and the plate was placed in a hybridization oven at 48°C for 2 min to dilute the probe.
  - Two hundred microliters of pre-heated solution 1 (48°C) was added per well, and the plate was placed in a hybridization oven at 48°C for 10 min; this was repeated once.
  - Two hundred microliters of pre-heated solution 2 (48°C) was added per well, and the plate was placed in a hybridization oven at 48°C for 20 min; this was repeated three times.
  - Two hundred microliters of PBT per well was added and the reaction plate was placed at room temperature (25°C) for 5 min; this was repeated once.
5. Staining: 1.8 mL → 200 μL/well
  - PBtr (1× PBS, 0.1% Triton-X 100, 0.1% BSA, 0.01% NaN_3_) and Staining Buffer (100 mM NaCl, 5 mM MgCl_2_, 100 mM Tris–HCl (pH 9.5), 0.1% Tween-20, and 1 mM levamisole) were prepared.
  - Two hundred microliters PBtr was added to each well and the reaction was carried out at room temperature (25°C) for 1.5 h.
  - Two hundred microliters of anti-DIG antibody solution (1,2000 with PBtr) was added to each well and the 8-well plate was placed in a humidity gasket and protected from light overnight at 4°C.
  - Two hundred microliters PBtr was added to each well and the reaction was repeated thrice for 10 min at room temperature (25°C).
  - Two hundred microliters of staining buffer per well was added and the reaction was repeated for 5 min at room temperature (25°C).
  - Two hundred microliters of coloring solution (NBT/BCIP) was added to each well and incubated at room temperature in the dark for ~4–8 h. Staining was observed every 1 h. Staining was terminated when most of the antisense probe set was stained.
  - Termination of staining: A mixture of 200 μL PBS and 0.2 M EDTA was added per well and reacted for 5 min at room temperature (25°C); this was repeated twice.
  - Imaging: An appropriate amount of nematode sample was taken on a glass slide, a coverslip was applied, the slide was sealed with glycerol, the slide was observed using a fluorescent upright optical microscope and photographs were taken to record the results.

#### 2.2.6 Cut-off method for PWN *Bx-vap-2* and *fem-2* whole gene and cut-off *in situ* hybridization

1. cDNA probe preparation
  - PCR amplification of plasmids was performed with a reaction system consisting of 0.5 μL template (plasmid), 1.0 μL each of forward and reverse primers, 1.0 μL dNTPs, 2.0 μL PCR buffer, 0.2 μL Taq polymerase, and 14.3 μL ddH_2_O. The reaction procedure was the same as that described in Section 2.2.3 (2). The PCR products were purified and detected by agarose gel electrophoresis.
  - Asymmetric PCR amplification of DIG-labeled sense- and antisense single-stranded DNA

Reaction system: 2.0 μL of purified PCR product, 2.0 μL of forward or reverse primer, 2.0 μL of PCR DIG-labeling mixture, 2.0 μL of PCR buffer, 0.2 μL of Taq polymerase, and 11.8 μL of ddH_2_O.

- PCR amplification products were detected using agarose gel electrophoresis, and DIG-labeled cDNA probes were stored at −20°C.

##### 2.2.6.1 Fixation of PWNs

1. Fixation of PWNs
  - The nematodes were washed three times with 1× PBS and fixed in 10% formaldehyde diluted with 1× PBS at room temperature for 2 days. After 2 days of nematode fixation, fixed nematodes were randomly cut on slides with a razor blade until approximately 90% of the nematodes were cut. The cut nematodes did not require repeated washing.
  - Nematodes were washed three times with 1× PBS, and nematode sections were partially digested with 500 μg/mL proteinase K for 1 h at room temperature (25°C).
  - The nematodes were washed once with 1× PBS, PBS was removed, and the nematodes were frozen at −80°C for 20 min.
  - The nematodes were resuspended in 1 mL of −20°C methanol, allowed to stand for 30 s on dry ice or at −80°C and then centrifuged at 16200× *g* for 30 s.
  - The nematodes were resuspended in 1 mL of acetone at – 20°C, incubated on dry ice or − 80°C for 1 min, and then centrifuged at 16200× *g* for 30 s.
  - Acetone was removed until approximately 100 μL remained in the tube, and 200 μL of ddH_2_O was then added to the tube.
  - The supernatant was removed and the nematodes were washed once with 1 mL of hybridization buffer, resuspended in 1 mL of hybridization buffer, and stored at −20°C.
2. Hybridization of PWNs
  - Nematode samples were aliquoted into 8-well plates, ensuring approximately 300 intact nematodes or 500 nematode sections per well, with three replicates at each age.
  - The DIG-labeled cDNA probes were denatured at 94°C for 2 min and cooled on ice.
  - Two microliters of antisense and sense (negative control) DIG-labeled cDNA probes were added to the appropriate wells. The 8-well plate was sealed with Parafilm, and the plate was placed on a rotator or shaker at 42°C overnight.
  - The plate was washed three times with 200 μL/well 4× SSC at 55°C for 15 min each.
  - The plate was then washed thrice with 200 μL/well of 0.1× SSC/0.1% SDS for 10 min each at 55°C.
3. Staining
  - The nematodes were washed once with 1× maleic acid buffer.
  - Nematodes were incubated in 1% Boehringer’s blocking reagent (Bbr, 10% stock solution diluted in maleic acid buffer) for 30 min.
  - The nematodes were then incubated with anti-DIG antibody (diluted 1:1000 with 1% Bbr) for 2 h.
  - The sample was washed thrice for 15 min each with 200 μL/well of 1× maleic acid buffer and 0.01% Tween-20.
  - The sample was then rinsed briefly in 1 mL 1× PBS.
  - Staining solution was prepared by adding 4.5 μL of NBT stock solution and 3.5 μL of BCIP to 1 mL of alkaline phosphatase assay buffer, and the nematodes were stained overnight in the dark (without agitation) at 100 μL/well.
  - The nematodes were washed twice with ddH_2_O containing 0.01% Tween-20 to stop the staining reaction.

## 3 Results and analysis

To clearly present a comparison of the results for the two *in situ* hybridization methods, we herein show a side-by-side comparison of the whole-mount and cut-off methods for whole PWNs as well as sections, on a gene-by-gene basis.

### 3.1 Results of *in situ* hybridization of *Bx-vap-2* and *fem-2* genes

**Figure 1:**
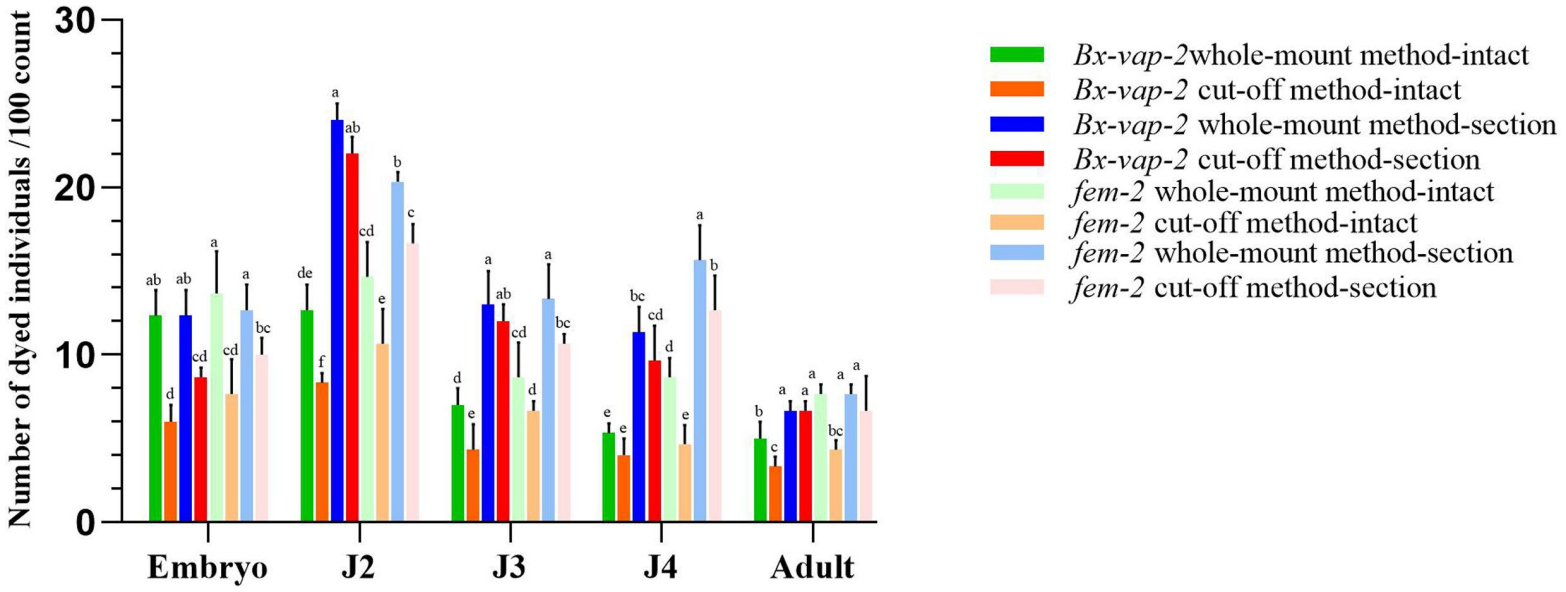
Results of *in situ* hybridization of *Bx-vap-2* and *fem-2* genes using two methods. The legend of different colors carries different meanings. Warm colors represent the data obtained through the whole-mount method, while cool colors represent the data obtained through the cut-off method. The bar chart in green, orange, blue, and red represents the data associated with the Bx-vap-2 gene. The light green, light orange, light blue, and light red bar chart represents the data related to the fem-2 gene. Means and ± SE from three biological replicates are shown. Different letters at the top of the bars represent statistically significant differences (one-way ANOVA; *p* < 0.05).

After performing *in situ* hybridization using different methods on the *Bx-vap-2* and *fem-2* genes of the pine wood nematode (PWN), a random 30 μL aliquot of the stained PWN sample was observed under a microscope. Staining numbers of the corresponding gene were counted for every 100 individuals or 100 sections and repeated thrice at each age to obtain the staining result data (Figure 1). The data presented in this study represent the putative staining sites or correctly expressed regions, as identified via analysis of the relevant literature.

In Figure 1, the legend of different colors carries different meanings. Warm colors represent the data obtained through the whole-mount method, while cool colors represent the data obtained through the cut-off method. The bar chart in green, orange, blue, and red represents the data associated with the *Bx-vap-2* gene, The light green, light orange, light blue, and light red bar chart represents the data related to the *fem-2* gene. From the graph, it can be observed that in various developmental stages of the pine wood nematode, regardless of the whole-mount or cut-off *in situ* hybridization methods, the overall staining intensity per 100 worms is generally higher in the whole-mount method (indicated by higher bars in the cool colors) compared to the cut-off method (indicated by lower bars in the warm colors). This suggests that the whole-mount method is more effective at staining compared to the cut-off method.

Furthermore, from the graph, we also observed that the hybridization efficiency of J2 stage is much higher than that of adults. This may be due to the fact that all developmental stages of pine nematode reproductive-stage nematodes are structurally very similar, except for the development of the gonads. The cuticle consists of four layers: a thin outer cortical layer (ECL), a homogeneous inner cortical layer (ICL), a middle layer (ML), and a striped basal layer (BL). However, as the nematodes grow, the thickness of the cuticle increases, from 0.19 μm in juveniles to 0.29 μm in adults (Kondo and Ishibashi, 1978). The thickening of the body wall makes it difficult for large molecular probes to enter, resulting in lower hybridization efficiency in adults compared to J2 stage.

The results indicated that the hybridization signal of intact individuals stained using the cut-off method was lower than that of individuals stained using the whole-mount method. The potential reasons for this discrepancy could be attributed to the shorter processing time in the severed method, which may result in incomplete digestion of the nematode cuticle barrier through physical and chemical treatments. Alternatively, it could be due to the cDNA probe being less capable of entering the nematode body or forming tight binding compared to the ssRNA probe. In the case of whole-mount *in situ* hybridization of pine wood nematode sections, the ssRNA probe appeared to bind to non-specific sites within the nematode sections. This could be attributed to the longer duration required for the whole-mount method or the higher propensity of the ssRNA probe to enter the nematode body, leading to non-specific hybridization signals.

### 3.2 Results of *in situ* hybridization of *Bx-vap-2* gene

**Figure 2:**
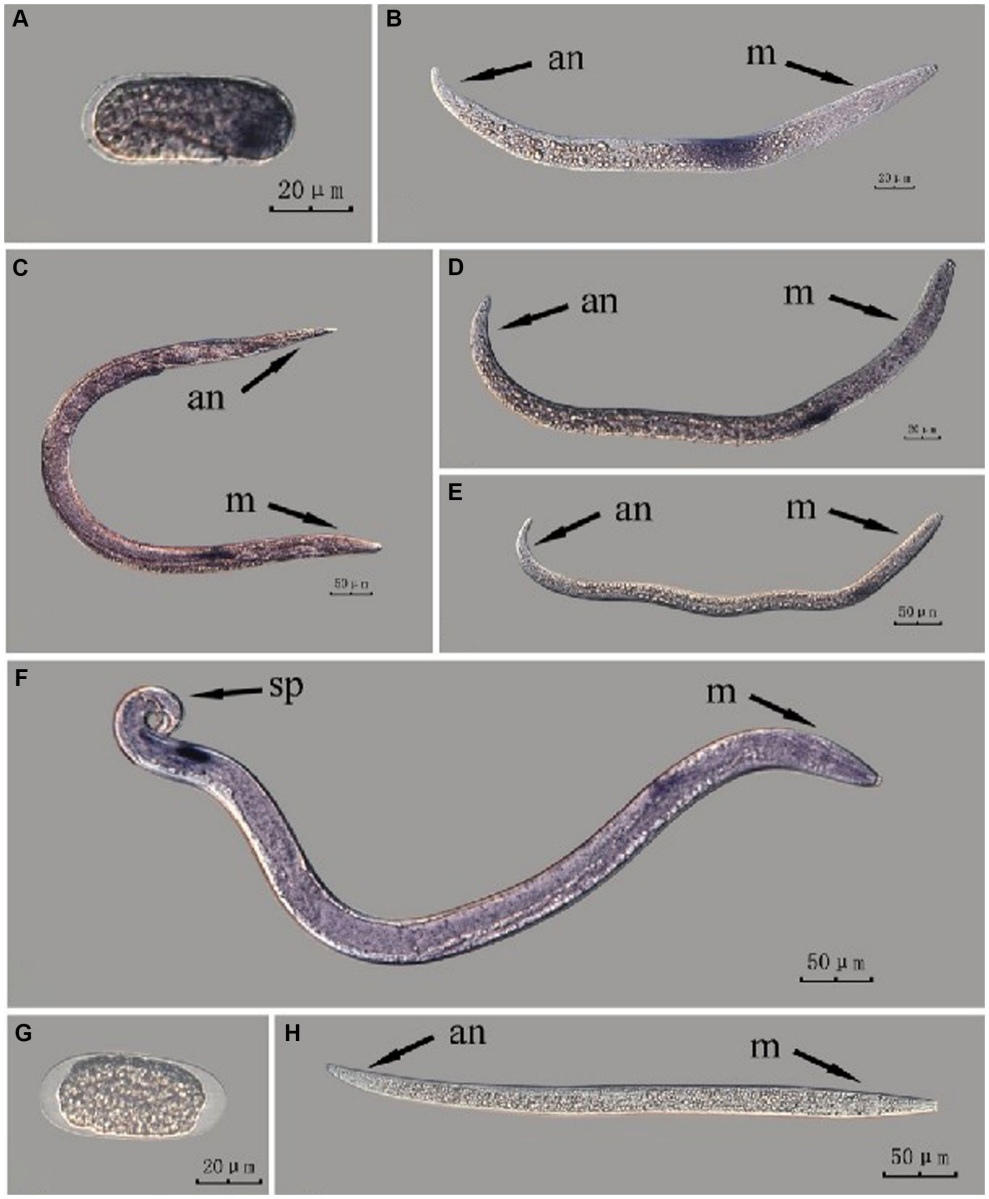
*In situ* hybridization of *Bx-vap-2* gene in different developmental stages of intact PWN (*Bursaphelenchus xylophilus*) using whole-mount method. A random 30 μL aliquot of the stained PWN sample was observed under a microscope and repeated thrice at each age. Representative photographs are shown. Panels **(A–F)** indicate PWN embryos, 2nd instar larvae, females, 3rd instar larvae, 4th instar larvae, and males, respectively; panels **(G,H)** represent unlabeled controls. m, median bulb; an, anus; sp., spicule.

**Figure 3:**
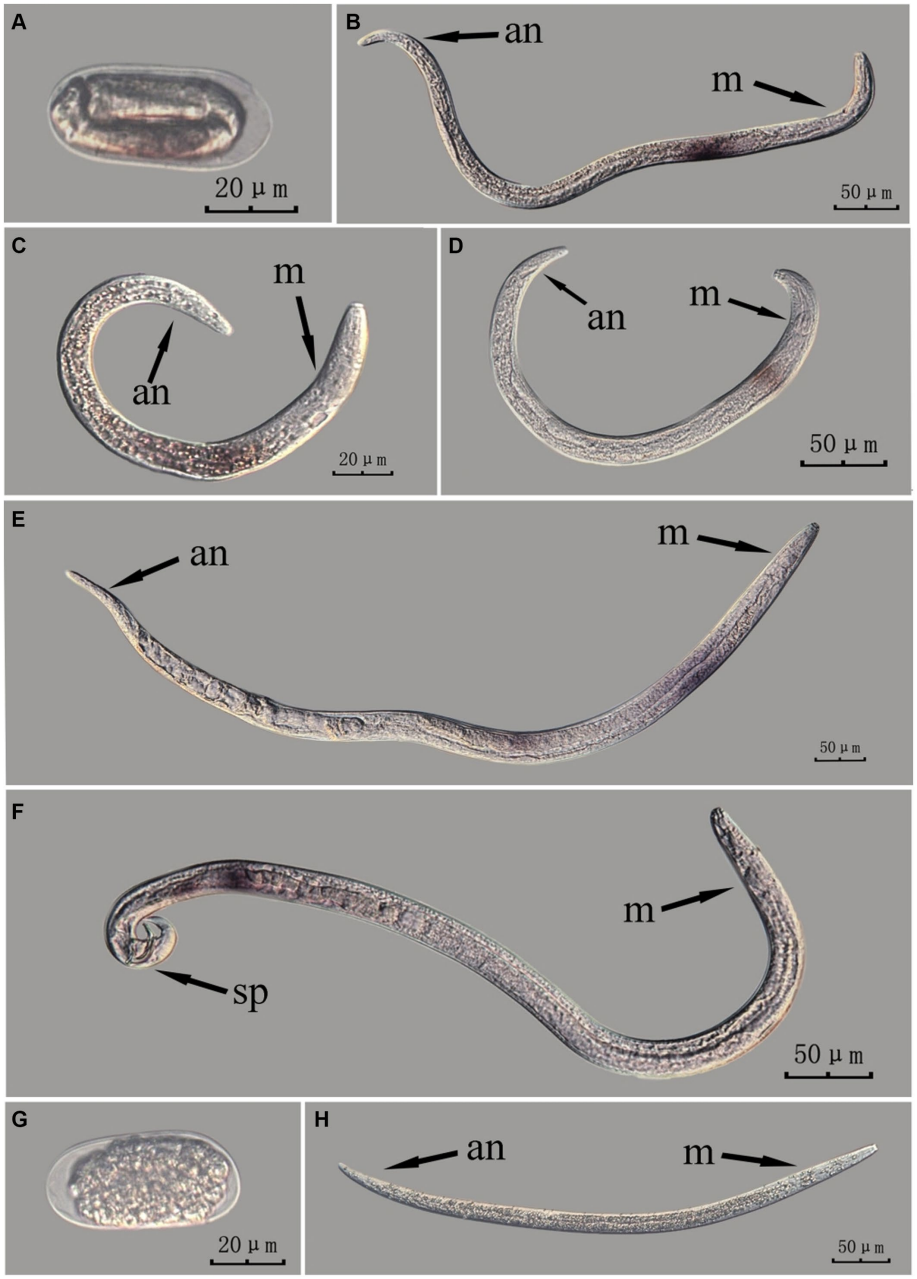
*In situ* hybridization of *Bx-vap-2* gene in different developmental stages of intact PWN (*Bursaphelenchus xylophilus*) using cut-off method. A random 30 μL aliquot of the stained PWN sample was observed under a microscope and repeated thrice at each age. Representative photographs are shown. Panels **(A–F)** indicate PWN embryos, 4th instar larvae, 2nd instar larvae, 3rd instar larvae, females, and males, respectively, and panels **(G,H)** represent unlabeled controls.

The *Bx-vap-2* gene in intact nematodes at different developmental stages was hybridized *in situ* by the methods of whole-mount and cut-off (Figures 2, 3).

**Figure 4:**
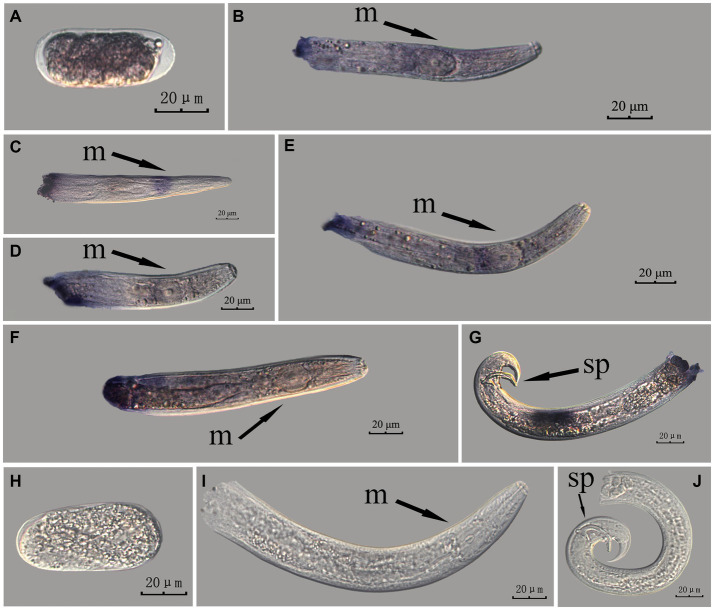
*In situ* hybridization of *Bx-vap-2* gene at different developmental stages of PWN sections (*Bursaphelenchus xylophilus*) using the whole-mount method. A random 30 μL aliquot of the stained PWN sample was observed under a microscope and repeated thrice at each age. Representative photographs are shown. Panels **(A–G)** indicate PWN embryos, 4th instar larvae, 2nd instar larvae, 3rd instar larvae, females, males, and males, respectively; and panels **(H–J)** represent unlabeled controls. m, median bulb; an, anus; sp., spicule.

**Figure 5:**
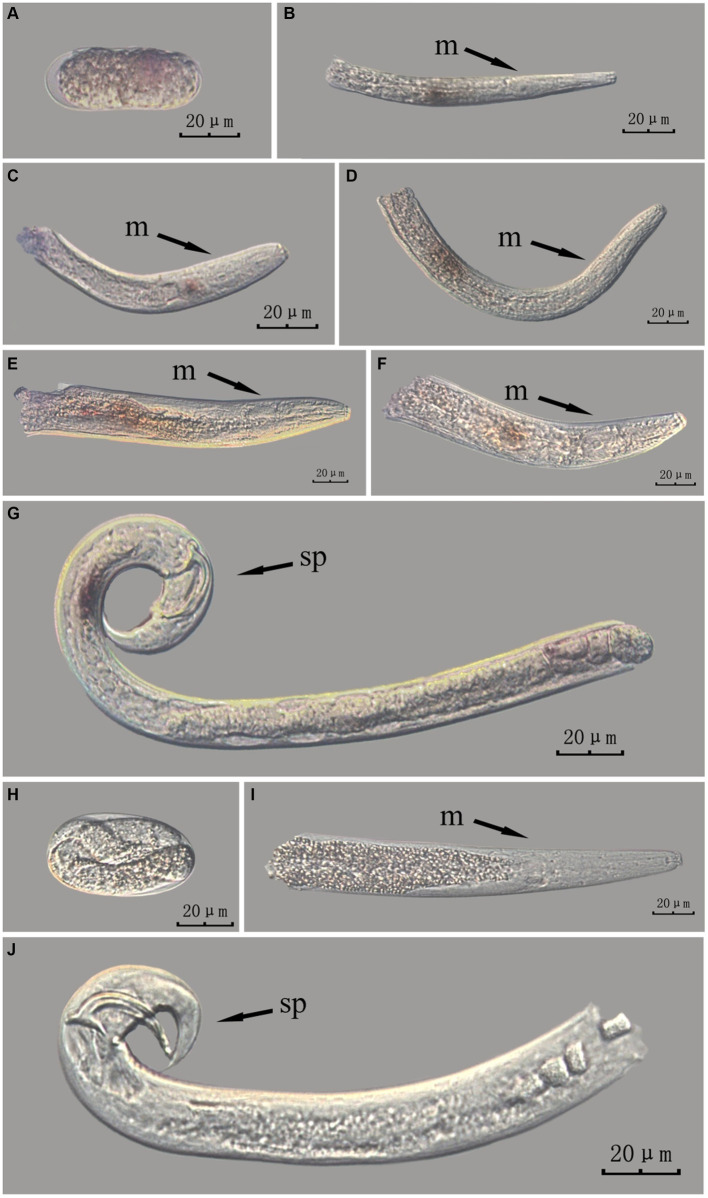
*In situ* hybridization of *Bx-vap-2* gene in different developmental stages of PWN sections (*Bursaphelenchus xylophilus*) using the cut-off method. A random 30 μL aliquot of the stained PWN sample was observed under a microscope and repeated thrice at each age. Representative photographs are shown. Panels **(A–G)** indicate PWN embryos, 3rd instar larvae, 2nd instar larvae, 4th instar larvae, females, males, and males, respectively, and panels **(H–J)** represent unlabeled controls. m, median bulb; an, anus; sp., spicule.

The *Bx-vap-2* gene in PWN sections at different developmental stages was hybridized *in situ* by the methods of whole-mount and cut-off methods (Figures 4, 5).

*In situ* hybridization experiments using the whole-mount method showed a blue-purple hybridization signal, whereas those using the cut-off method showed a reddish-brown hybridization signal.

This paragraph describes the results of performing *in situ* hybridization of the *Bx-vap-2* gene using the whole-mount method in different developmental stages of intact PWN. Espada et al. (2016) discovered that a predicted VAP protein (*BUX.s00116.606*) is expressed in the subventral gland cells (Espada et al., 2016). The experimental results in this section indicate that the *Bx-vap-2* gene exhibits patchy expression in the J2 stage. The hybridization signal is primarily distributed in the middle region of the J2 body and is not precisely localized near the esophageal gland area. In the J3, J4, and adult stages, the hybridization signals were primarily expressed in stripes, with the expression sites in the anterior esophageal gland of nematodes predominantly observed during the J4 and adult stages. This pattern closely corresponds to the synthesis of the *Bx-vap-2* gene within the esophageal gland and its secretion through the needle-like stylet at the nematode’s mouth. However, the hybridization signals in J3 were found to be distant from the vicinity of the esophageal gland. A hybridization signal was also identified in the gonads anterior to the male spicules, and it is speculated that the *Bx-vap-2* gene may be associated with male reproduction. Based on the experimental results in this section, the *in situ* hybridization results for J2 and J3 stages were not sufficiently precise, indicating that the overall accuracy of this method, in terms of locating pathogenic genes, is relatively low (Figure 2).

This paragraph describes the results of performing *in situ* hybridization of the *Bx-vap-2* gene using the cut-off method in different developmental stages of intact PWN. The *Bx-vap-2* gene was expressed in small patches at different developmental stages of the PWN, and its expression was mainly at the location of the esophageal gland at the anterior end of the worm. However, because the incubation time of the cut-off method was shorter than that of the whole-mount method, or because the cDNA probe could not easily penetrate the body wall, the hybridization signal intensity for the *Bx-vap-2* gene of the intact PWN in the cut-off *in situ* hybridization was less than that of the whole-mount method (Figure 3). Additionally, the *in situ* hybridization signals in J4 (Figure 3B) were not precisely observed in the esophageal gland area of the nematode, suggesting that the utilization of the cut-off method, including probes, reagents, and techniques, on intact nematodes did not improve the accuracy of gene localization.

This paragraph describes the results of performing *in situ* hybridization of the *Bx-vap-2* gene using the whole-mount method in different developmental stages of PWN sections. The *Bx-vap-2* gene showed indistinct punctate expression at different developmental stages of the PWN, and the hybridization signal at the J2 stage was mainly distributed at a slightly anterior position to the median bulb. We suspected it to be related to the secretion of the *Bx-vap-2* gene. Deeper staining was observed at the cut-off site, except for eggs; this may be related to the non-specific binding of the ssRNA probe at the cut-off sites (Figure 4).

This paragraph describes the results of performing *in situ* hybridization of the *Bx-vap-2* gene using the cut-off method in different developmental stages of PWN sections. The *Bx-vap-2* gene showed distinctive punctate expression in eggs, J2, J3, and J4 larvae, and males, and striped expression in females. Compared with those labeled in the whole-mount method, the sections in the cut-off method hardly produced any false hybridization signals at the cut-off sites. Based on the staining results, the labeling of the nematode sections via the cut-off method was better than that of the whole-mount method, with a clearer distribution of the hybridization signals (Figure 5).

Based on previously published papers on pathogenic genes of the pine wood nematode, Espada et al. (2018) validate that a subset of STATAWAARS-containing genes are specifically expressed in the pharyngeal glands. Zhang et al. (2022) observed strong signals of three specific legumains (*BxCN10334, BxCN10337*, and *BxCN10284*) in the esophageal glands of both juvenile and adult PWN using digoxigenin-labeled antisense cDNA probes. Qiu et al. (2023) used a digoxigenin-labeled *BxLip-3* antisense complementary DNA (cDNA) probe and the results demonstrated specific expression of *BxLip-3* in the dorsal gland and intestine of PWNs. *In situ* hybridization experiments during the embryonic stage were deemed unnecessary. The purpose of this study was to compare the effectiveness of these two *in situ* hybridization methods. Based on the experimental results, neither of the *in situ* hybridization methods proved suitable for localizing pathogenic genes during the embryonic stage of the pine wood nematode. Non-specific hybridization signals were prone to occur.

### 3.3 Results of *in situ* hybridization of *fem-2* gene

**Figure 6:**
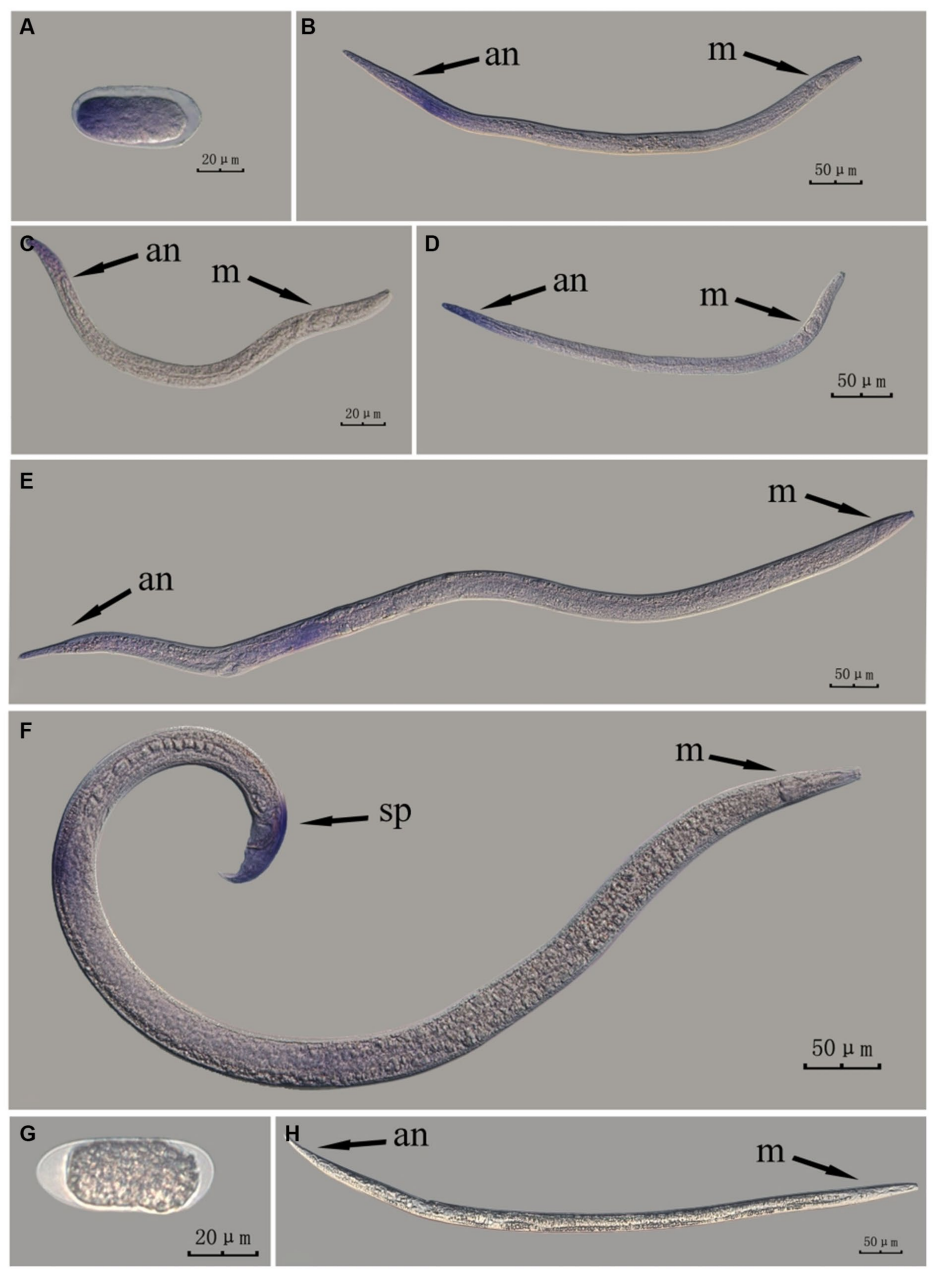
*In situ* hybridization of *fem-2* gene in different developmental stages of intact PWN (*Bursaphelenchus xylophilus*) using whole-mount method. A random 30 μL aliquot of the stained PWN sample was observed under a microscope and repeated thrice at each age. Representative photographs are shown. Panels **(A–F)** indicate PWN embryos, 4th instar larvae, 2nd instar larvae, 3rd instar larvae, females, and males, respectively, and panels **(G,H)** represent unlabeled controls. m, median bulb; an, anus; sp., spicule.

**Figure 7:**
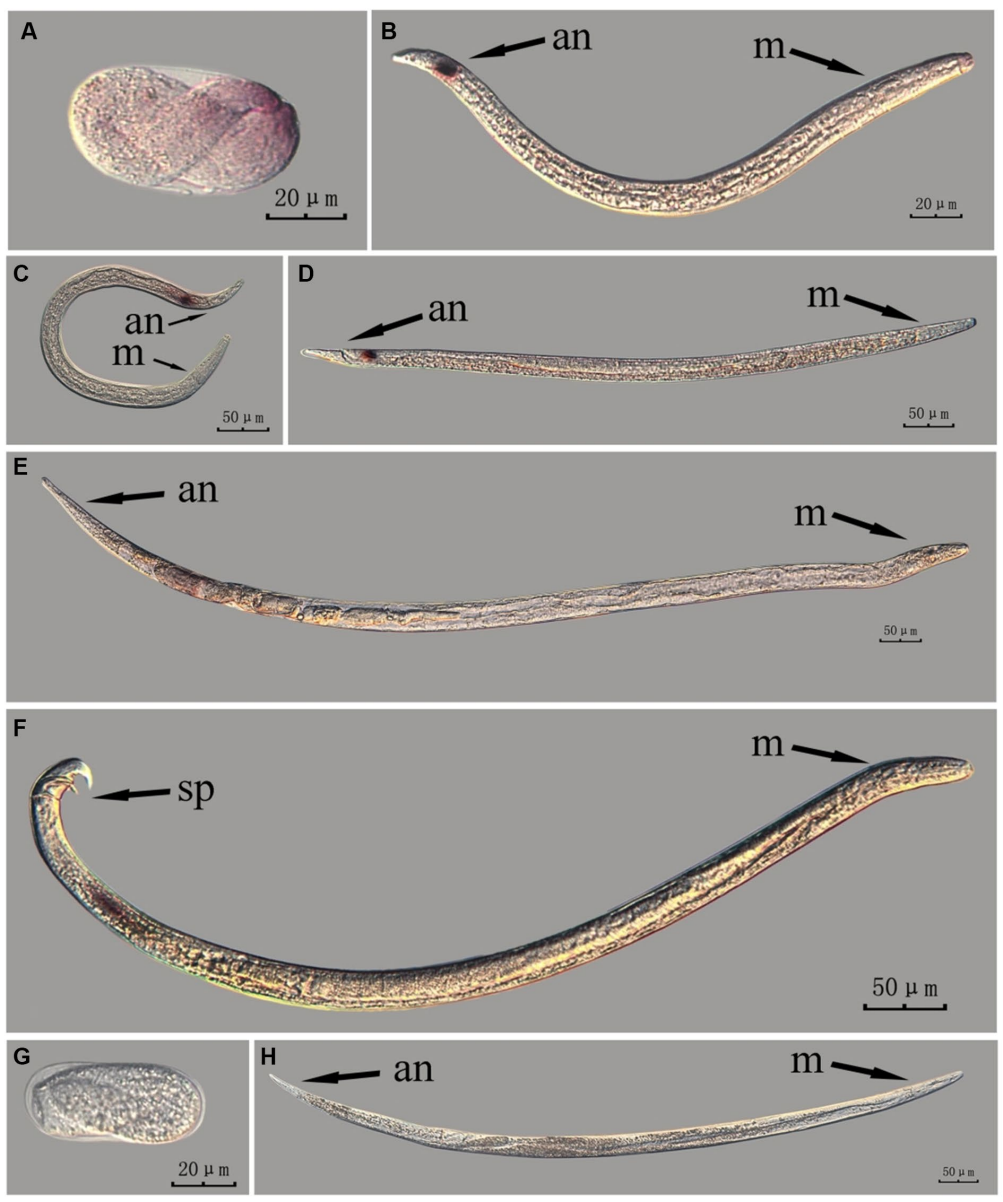
*In situ* hybridization of *fem-2* gene in different developmental stages of intact PWN (*Bursaphelenchus xylophilus*) using the cut-off method. A random 30 μL aliquot of the stained PWN sample was observed under a microscope and repeated thrice at each age. Representative photographs are shown. Panels **(A–F)** indicate PWN embryos, 2nd instar larvae, 3rd instar larvae, 4th instar larvae, females, and males, respectively, and panels **(G,H)** represent unlabeled controls. m, median bulb; an, anus; sp., spicule.

The *fem-2* gene in intact nematodes at different developmental stages of PWN was hybridized *in situ* by the methods of whole-mount and cut-off (Figures 6, 7).

**Figure 8:**
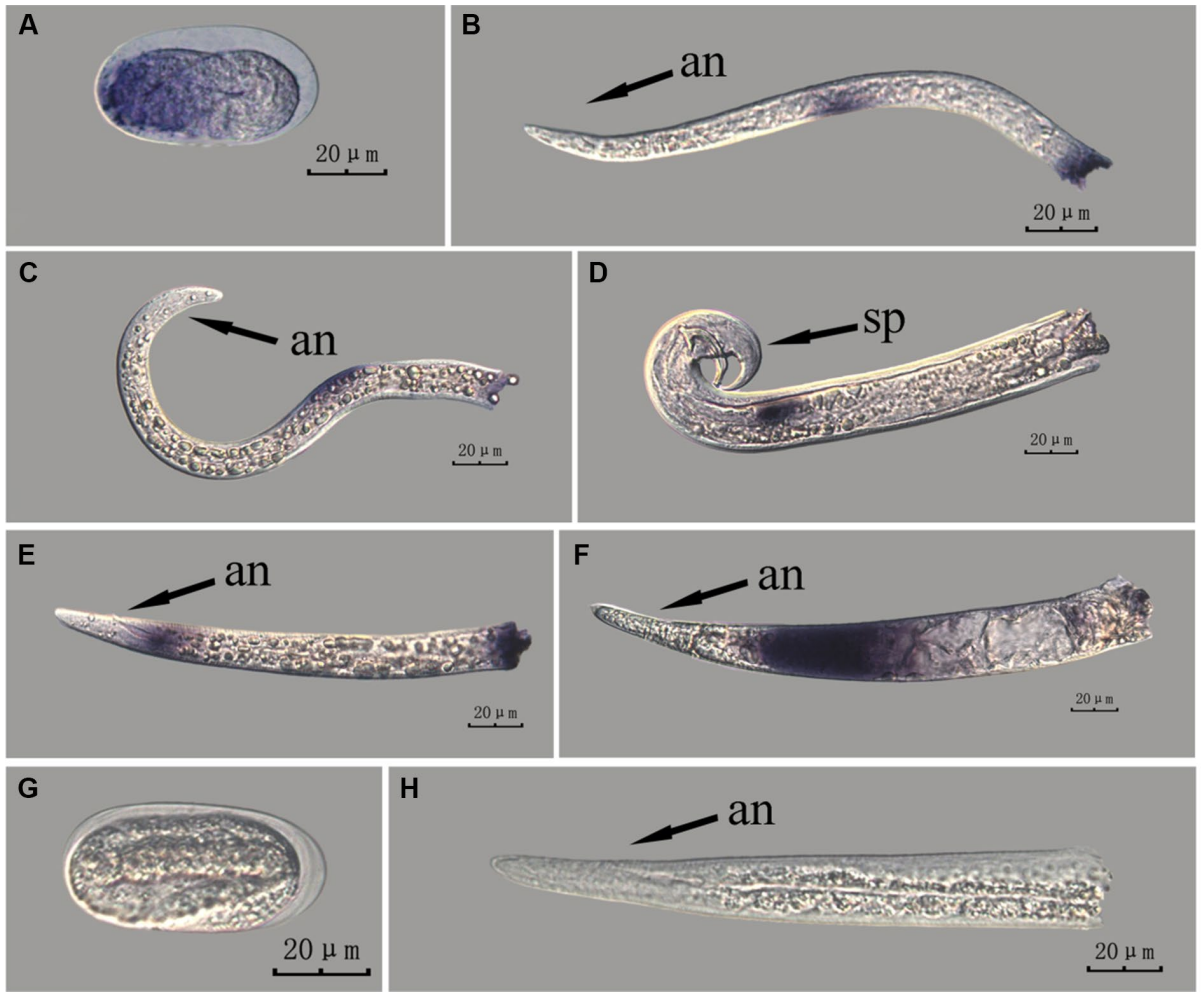
*In situ* hybridization of *fem-2* gene at different developmental stages of PWN sections (*Bursaphelenchus xylophilus*) using the whole-mount method. A random 30 μL aliquot of the stained PWN sample was observed under a microscope and repeated thrice at each age. Representative photographs are shown. Panels **(A–F)** indicate PWN embryos, 2nd instar larvae, 3rd instar larvae, males, 4th instar larvae, and females, respectively, and panels **(G,H)** represent unlabeled controls. m, median bulb; an, anus; sp., spicule.

**Figure 9:**
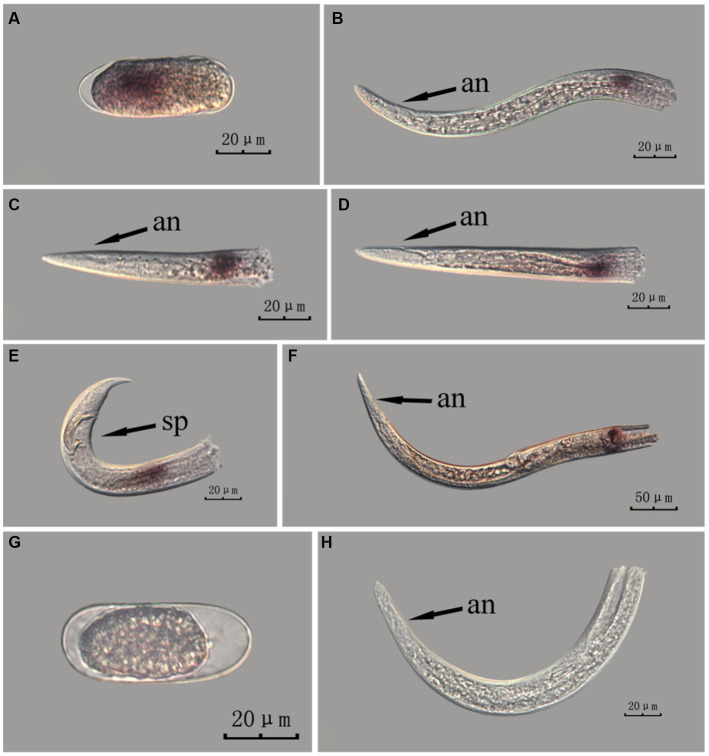
*In situ* hybridization of *fem-2* gene in different developmental stages of PWN sections (*Bursaphelenchus xylophilus*) using the cut-off method. A random 30 μL aliquot of the stained PWN sample was observed under a microscope and repeated thrice at each age. Representative photographs are shown. Panels **(A–F)** indicate PWN embryos, 4th instar larvae, 2nd instar larvae, 3rd instar larvae, males and females, respectively, and panels **(G,H)** represent unlabeled controls. m, median bulb; an, anus; sp., spicule.

The *fem-2* gene in PWN sections at different developmental stages was hybridized *in situ* by the methods of whole-mount and cut-off methods (Figures 8, 9).

The *fem-2* gene was expressed differently at each developmental stage of PWN in both methods.

This paragraph describes the results of performing *in situ* hybridization of the *fem-2* gene using the whole-mount method in different developmental stages of intact PWN. The *fem-2* gene was expressed in patches at different developmental stages of the PWN, and the hybridization signal was mainly distributed in the tail part of the nematode at the J2 stage, which was presumably related to the formation of papillae and other structures. At the J3 and J4 stages, the expression was mainly observed at the tails. In females, the hybridization signal was mainly distributed in the ovaries, whereas in males, it was mainly distributed in the spicule and testis. This is consistent with *fem-2* gene as a sex-determining gene (Figure 6).

This paragraph describes the results of performing *in situ* hybridization of the *fem-2* gene using the cut-off method in different developmental stages of intact PWN. The *fem-2* showed patched expression in eggs and females, whereas its expression was distinctly punctate in the J2, J3, and J4 stages. It was expressed in stripes in the testis of males. Based on the staining results for the *fem-2* gene, intact nematodes in the whole-mount method appeared to display gene expression of varying intensity of hybridization signal patches, whereas intact nematodes in the cut-off method exhibited a more intense punctate hybridization signal (Figure 7). The development of gender-related gland is a continuous process, and while cut-off method clearly identifies the hybridization sites via point signals, it is less effective in illustrating the continuous developmental process compared to the whole-mount method.

This paragraph describes the results of performing *in situ* hybridization of the *fem-2* gene using the whole-mount method in different developmental stages of PWN sections. The *fem-2* gene showed patched expression in eggs, J2 and J3 stages, and females, but punctate expression in J4 stage and males. The whole-mount method inevitably produced varied hybridization signals at the cut-off sites, except for eggs (Figure 8).

This paragraph describes the results of performing *in situ* hybridization of the *fem-2* gene using the cut-off method in different developmental stages of PWN sections. The *fem-2* gene showed punctate expression at different developmental stages in PWN. Compared to the PWN sections in the whole-mount method, those produced by the cut-off method produced fewer false hybridization signals at the cut-off sites. Based on our results, the staining effect of the PWN sections in the cut-off method was slightly better than that of the whole-mount method, with a clearer clearer hybridization signal position (Figure 9).

The hybridization signals indicate that the whole-mount method and the cut-off method yield different localization results for *fem-2* in J3 and J4 stage larvae. This discrepancy may be attributed to the fact that gender differentiation in the pine wood nematode occurs only in the late J4 stage, and thus, the J3 and J4 stages cannot be distinguished in terms of gender in the experimental results. At this stage, the reproductive glands of the pine wood nematode are in the developmental phase, and the developmental status may vary among individuals. The inconsistent development of the reproductive glands leads to the aforementioned experimental results.

In Figures 6–9 of the paper, the differences in hybridization efficiency of pine wood nematode eggs may be due to variations in the two different *in situ* hybridization methods used, or it could be attributed to the eggs being in different developmental stages. If *in situ* hybridization methods are to be used to study genes related to development, we suggest collecting nematode embryos at different developmental stages for experimentation, in order to obtain more accurate experimental results.

### 3.4 Digital analysis of the *in situ* hybridization results

In this study, the *in situ* hybridization of *Bx-vap-2* and *fem-2* genes was performed using both the whole-mount and cut-off method. The results were compared in terms of staining rate, correct staining rate, experiment time, and operation difficulty (Table 1). Staining rate is the apparent number of staining statistics, and correct staining rate is the rate of staining based on the analysis of possible staining sites according to the literature of related genes (Lin et al., 2011; Chen et al., 2021). Calculation method: count the staining of the corresponding gene for every 100 articles or 100 segments, and repeat three times for each age.

**Table 1:** Comparison of two *in situ* hybridization methods based on digital analysis.

Evaluation criterion	Whole-mount ISH	Cut-off ISH
Staining rate	32%	27%
Correct staining rate	12%	9%
Experiment time	11 h	6 h
Operation difficulty	Easier	Easy

The staining rate refers to the apparent staining quantity, while the accurate staining rate refers to the staining rate that was calculated based on the possibly stained site analysis according to the relevant gene literature.

## 4 Discussion and conclusion

This study investigated the most suitable *in situ* hybridization method for studying PWN using the localization of two distinctive genes. After hatching, PWNs have two distinct life stages: a larval stage for feeding and dispersal, and an adult stage for mating and reproduction. Because the two stages of PWNs are morphologically and physiologically very different, gene expression in these stages is also very different (Mamiya, 1975). It has been shown that PWN secretions such as cellulases, peroxidases, proteases, and amylases play an important role in pathogenesis; the *Bx-vap* gene family is associated with esophageal gland secretions (Lin et al., 2011). In the reproductive stage, sex ratio is an important factor that affects the PWN reproductive rate (Zhou et al., 2021); the genes related to sex determination, including the *tra* and *fem* gene families, play an important role in this stage (Zarkower, 2006).

The study revealed that *Mimsp1* is the first identified *vap* gene in plant-parasitic nematodes, and it exhibits expression during the infection stage in second-stage larvae, suggesting its involvement in the nematode infection process (Ding et al., 2000). Additionally, Lin et al. detected the sequencing and mRNA expression of *Bx-vap-1*, *Bx-vap-2*, and *Bx-vap-3* genes, indicating their potential synthesis in the esophageal gland and secretion from the nematode’s oral cavity (Lin et al., 2011). Furthermore, the study provided further insights into the developmental expression localization of the *Bx-vap-2* gene in different stages of pine wood nematode. The *fem* gene has been closely associated with male nematode development and was initially identified in *Caenorhabditis elegans* (Doniach and Hodgkin, 1984). Chen et al. found that the *fem-1* and *fem-3* genes, belonging to the *fem* gene family, are primarily expressed in the gonads, intestine, and body walls, and play a role in the regulation of sex determination in pine wood nematodes (Chen et al., 2021). Given that both *in situ* hybridization methods used in this study localized the expression of the *fem-2* gene in reproductive organs such as the gonads, spicule sheath, and muscles near the copulatory spicule, the *in situ* hybridization results of this study are considered reliable.

To summarize the whole-mount and cut-off *in situ* hybridization methods in terms of experimental time, the probes used for the whole-mount method must be prepared 1 day in advance, and the *in situ* hybridization experiment took at least 3 days from start to finish; especially on the first day, the digestion and fixation pre-experiments took approximately 7 h, and the overall experiment time was approximately 11 h. In contrast, the probes for the cut-off method did not require advance preparation and could be combined with the pre-experiment on the first day. The cut-off *in situ* hybridization experiment took at most 3 days from start to finish, and the overall time of the experiment was approximately 6 h, which was approximately half the time compared to the whole-mount method, with flexible scheduling of experiment procedures. The staining counts per 100 individuals were greater for the whole-mount method than that for the cut-off method, indicating that PWNs were stained more easily using by the whole-mount method. The difference in staining counts may have been due to the fact that cDNA probes are less permeable or bind less tightly to targets than ssRNA probes. In terms of sample loss, both the whole-mount and cut-off methods are prone to the loss of PWN sections because, during the centrifugation and buffer transfer steps, partial nematode fragments did not precipitate by centrifugation.

After comparing two *in situ* hybridization methods for the application of two genes, it was observed that for the *fem-2* gene, the whole-mount method demonstrated a staining rate of 34% and an accurate staining rate of 12.5%. In contrast, the cut-off method showed a staining rate of 29.3% and an accurate staining rate of 9.3%. Similarly, for the *Bx-vap-2* gene, the whole-mount method exhibited a staining rate of 30.1% with an accurate staining rate of 10.9%. Conversely, the cut-off method yielded a staining rate of 25.8% and an accurate staining rate of 8.5%. In brief, the whole-mount method exhibited higher staining rates and correct staining rates for both the *fem-2* gene and the *Bx-vap-2* gene compared to the cut-off method. Based on the *in situ* hybridization staining results from the *fem-2* gene and *Bx-vap-2* gene, it can be observed from the images that the cut-off method yields better staining effects on pinewood nematode sections compared to the whole-mount method. The hybridization signals are more clearly localized, and there is less non-specific staining. In other words, the cut-off method demonstrated more precise localization of genes. Both methods are applicable for gene localization, but based on staining pattern, experimental results analysis, and comprehensive experimental operation, the whole-mount method is considered more appropriate for the localization and expression analysis of developmental-related genes in PWNs. Because intact pinewood nematodes are more conducive to showcasing the continuous developmental process of development-related genes. On the other hand, considering the experimental time, accuracy of staining site, and the amount of non-specific staining, the cut-off method is more fitting for pathogenic-related genes. In addition, for better performance, the cut-off method can be selectively applied to specimens during the experimentation.

In this study, the pathogenicity-related *Bx-vap-2* gene and the sex determination-related *fem-2* gene were selected to investigate the characteristics of *in situ* hybridization using whole-mount and cut-off methods, respectively, in PWN. The experimental findings demonstrate that the whole-mount approach is better suited for identifying genes associated with the development of pinewood nematodes, while the cut-off approach is more appropriate for locating genes relevant to pathogenicity. Moreover, researchers may tailor the cut-off approach to make necessary cuts to the nematode during experimentation based on the specific experimental context. This study also provides a relevant experimental method for *in situ* hybridization of other plant-parasitic nematodes, along with guidance as to which method would be most appropriate for conducting *in situ* hybridization experiments on targeted genes. We hope that this information will assist other researchers in avoiding unnecessary experimental design, ultimately conserving valuable time and resources.

## Data availability statement

The original contributions presented in the study are included in the article/Supplementary material, further inquiries can be directed to the corresponding author.

## Author contributions

KG and XZ: conceptualization. CW and ST: performing the experiments. CW, ST, and KG: writing—original draft preparation. XZ and LZ: writing—review and editing. JZ: writing – review and editing, methodology. All authors have read and agreed to the published version of the manuscript.
