# Supplementary material for: Comparative effects of two in situ hybridization methods for the pinewood nematode (Bursaphelenchus xylophilus)
Source: Front Microbiol. 2023 Nov 30;14:1234895. doi: 10.3389/fmicb.2023.1234895 (PMC10720641; doi:10.3389/fmicb.2023.1234895)
Supplement: Supplementary file 1 [file Table_1.docx]

Supplemental Table 1

| Reagents | Temperature | Time |
| --- | --- | --- |
| MeOH | 4°C | 5 min |
| MeOH:0.2 N HCl = 1:1 | 4°C | 10 min |
| 1× PBS | 4°C | 2 min |
| 1× PBS | 22°C | 5 min |
| 990 µL 1× BO_3_, 10 µL 1 M DTT | 22°C | 10 min |
| 1× BO_3_ | 22°C | 3 min, 2 min, 2 min, 2 min, 2 min |
| 980 µL 1× BO_3_, 20 µL 30% H_2_O_2_ | 22°C | 10 min |
| 1× PBS | 22°C | 2 min, 2 min, 2 min |
| 3.7% formaldehyde in HEPES-PBS | 22°C | 2 h |
