# Supplementary material for: Comparative effects of two in situ hybridization methods for the pinewood nematode (Bursaphelenchus xylophilus)
Source: Front Microbiol. 2023 Nov 30;14:1234895. doi: 10.3389/fmicb.2023.1234895 (PMC10720641; doi:10.3389/fmicb.2023.1234895)
Supplement: Supplementary file 2 [file Table_2.docx]

Supplemental Table 2

| Reagents | Temperature | Time |
| --- | --- | --- |
| PBT | 25°C | 5 min |
| 50% formamide, 5× SSC, 100 μg/mL heparin, 0.1% Tween:PBT = 1:1 | 25°C | 10 min |
| 50% formamide, 5× SSC, 100 μg/mL heparin, 0.1% Tween | 25°C | 10 min |
